# A Rare Location of a Repeat Ectopic Pregnancy: A Case Report

**DOI:** 10.7759/cureus.15982

**Published:** 2021-06-28

**Authors:** Madhu Singh, Rahul Singh, Abhishek B Singh

**Affiliations:** 1 Obstetrics and Gynecology, Dr. Balwant Singh's Hospital, Georgetown, GUY; 2 Accident and Emergency, Dr. Balwant Singh's Hospital, Georgetown, GUY; 3 Internal Medicine, Dr. Balwant Singh's Hospital, Georgetown, GUY

**Keywords:** sonography in the diagnosis of ectopic pregnancy, repeat ectopic, stump of tube, laparoscopic, hemoperitoneum, beta hcg

## Abstract

Ectopic pregnancies often recur in the same patients as its etiology is tubal damage, which is rarely unilateral. In the setting of a well-documented history of ectopic pregnancy in the past where a salpingectomy was performed, and the clinical picture now suggests another ectopic, it can be reasonably concluded that the ectopic is in the remaining tube. In the case we present here, the ultrasound findings also suggested a tubal pregnancy in the remaining tube. Therefore, it was a surprise to find a profusely bleeding ectopic gestation in the stump of the same tube (where salpingectomy was performed earlier) on laparoscopy.

## Introduction

An ectopic pregnancy occurs when an embryo implants and grows outside the uterine cavity. It is estimated to account for 1-2% of all pregnancies [1]. An ectopic pregnancy is one of the most common emergencies encountered in a gynecology unit. When diagnosed early, they may be managed medically [1]. However, when a tubal rupture occurs, and there is evidence of significant hemoperitoneum, or the patient is clinically unstable, an emergency diagnostic laparoscopy or laparotomy is mandated [2], depending on the capability of the treating center, along with a salpingectomy or a salpingostomy.

We report a case where the second ectopic occurred in the remaining stump of the previously removed fallopian tube in a patient presenting for the second time at our center. The importance of removing the entire tube when a salpingectomy was performed was apparent in this case.

## Case presentation

Our patient had a diagnosis of pregnancy by a positive urine human chorionic gonadotropin (hCG) at six weeks amenorrhoea. She had an ultrasound exam on the same day, which showed a thickened endometrium and no intrauterine or extra-uterine pregnancy. She was advised to consult with the gynecologist but did not comply. She presented again 10 days later with an acute abdomen, dizziness, and a history of syncope. She was advised to undergo a repeat ultrasound examination, which was suggestive of a massive hemoperitoneum and a tubal pregnancy on the right side. Significantly, this patient had a history of a ruptured ectopic pregnancy in the left tube five months prior to this presentation, for which she had undergone a laparoscopic left salpingectomy at that time.

**Figure 1:**
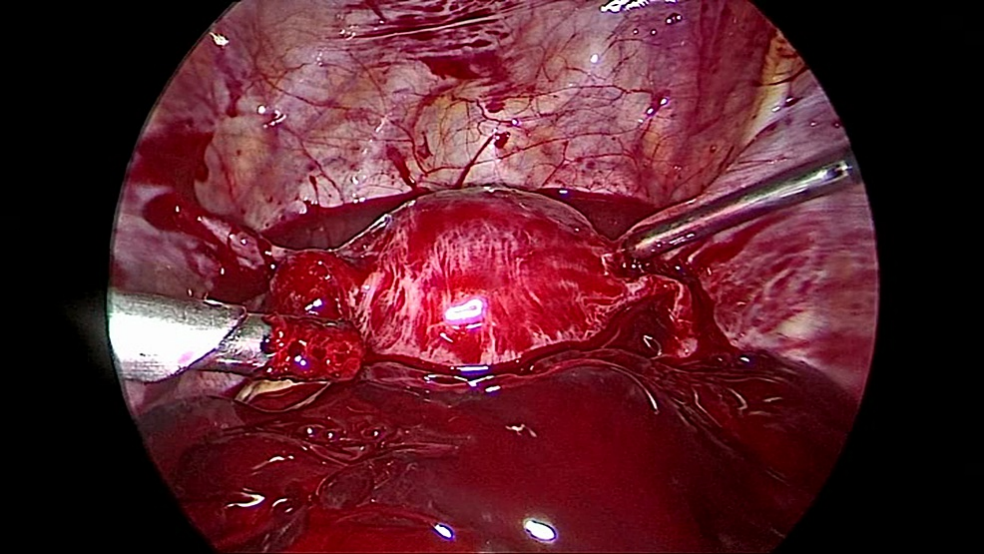
The view of the pelvis on insertion of the telescope showing blood and clots

As she had signs of hemoperitoneum and was unstable with tachycardia (heart rate of 106 beats per minute), hypotension (blood pressure of 90/50 mmHg), and was extremely pale, an emergency laparoscopy was planned and performed. On laparoscopy, a massive hemoperitoneum was found with blood and clots in the pelvis (Figure 1), anterior to and above the liver, next to the spleen, and in the paracolic gutters. Blood and clots were estimated to be about 2000 ml.

**Figure 2:**
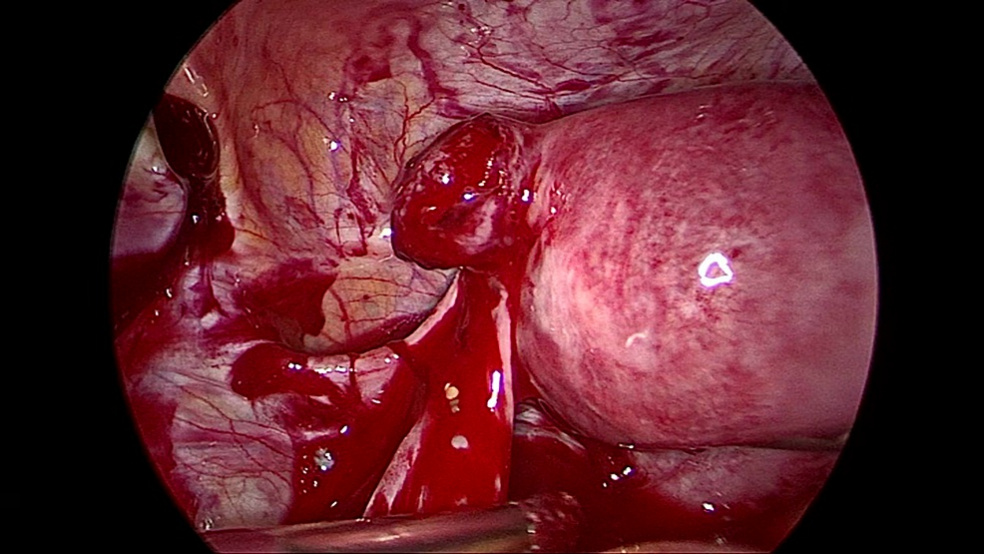
On performing suction, the ectopic was identified in the stump of the left tube, which was bleeding profusely

After performing preliminary suction, we were able to identify the source of the bleeding, which turned out to be the residual stump of the left tube. There was brisk bleeding from this stump (Figure 2).

**Figure 3:**
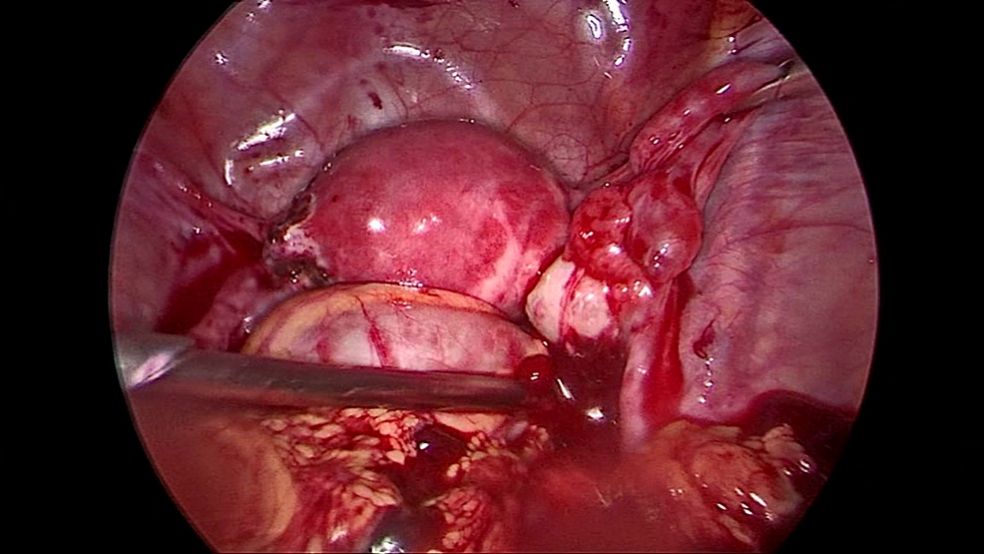
A few insignificant cysts were seen on the right side (paratubal)

The cornua of the uterus at the point of attachment of the left tube appeared edematous and congested. The right tube was normal and had a few small insignificant cysts in the mesosalpinx (Figure 3).

**Figure 4:**
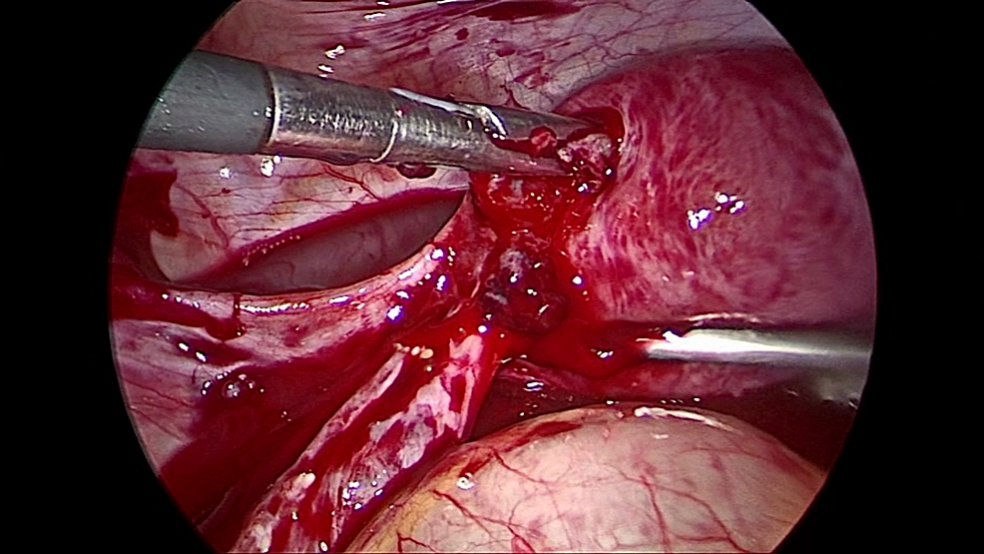
Chorionic tissue is seen being extruded from the stump

As the patient was unstable, instead of attempting a full cornual resection, we coagulated the stump that was actively bleeding with a bipolar sealer, and, in the process, the ectopic gestation sac was extruded from the stump (Figure 4).

**Figure 5:**
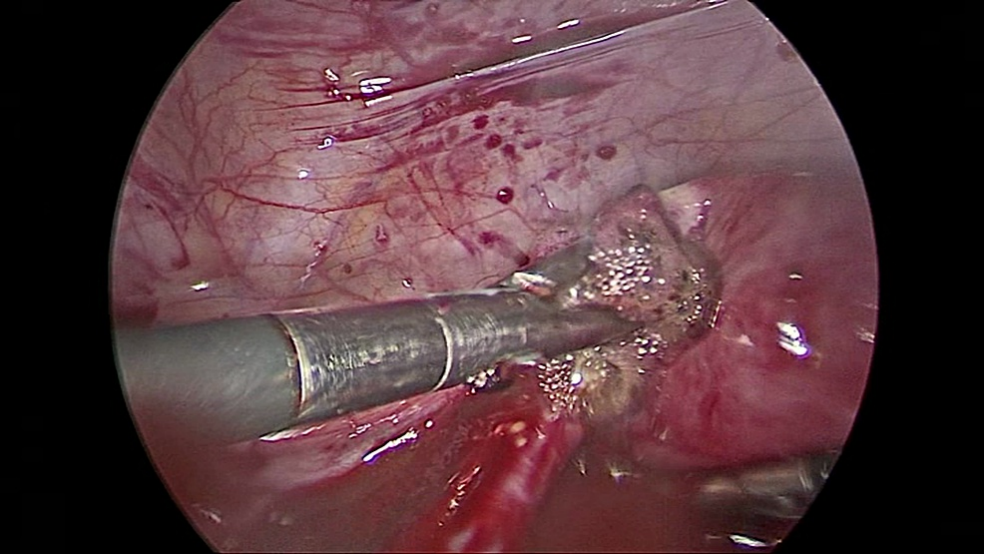
Bipolar coagulation of the stump using a bipolar sealer

**Figure 6:**
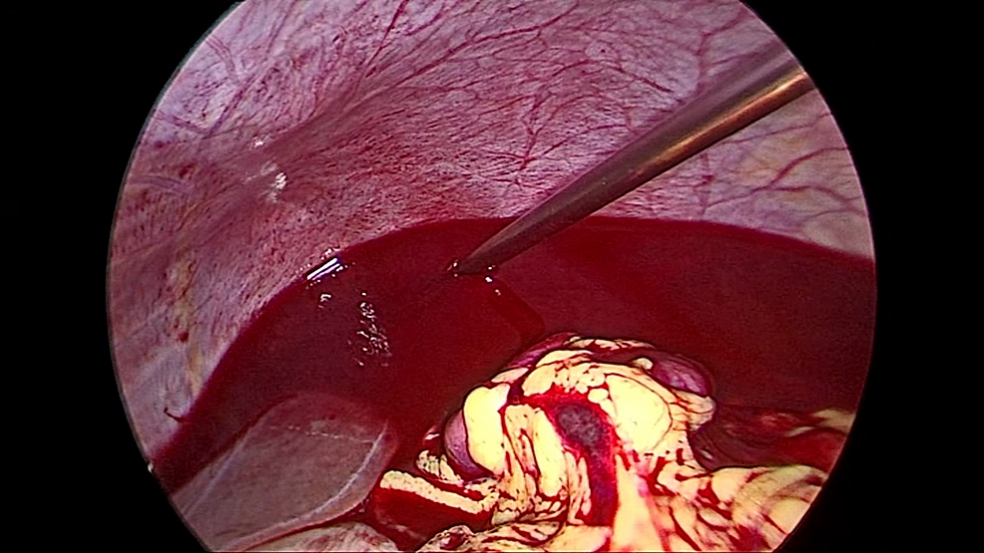
The image shows the magnitude of the hemoperitoneum, which extended above the liver

The stump was thoroughly coagulated using a bipolar sealer and the bleeding stopped entirely (Figure 5). Thorough lavage and suction were done for the hemoperitoneum (Figure 6).

A drain was placed in the pouch of Douglas and connected to a gravity drainage bag. The laparoscopic ports were closed. The patient was transfused a unit of compatible packed cells after surgery. She recovered well postoperatively and was discharged 48 hours after the surgery. We repeated a beta hCG a week after surgery, and the level was found to be declining satisfactorily.

Investigations

Urine hCG was done on March 6, 2021, after amenorrhoea of six weeks, and it was positive. Ultrasound examination on the same day showed a thickened endometrium. No intrauterine or extra-uterine pregnancy was seen. There was no free fluid, and the patient was advised to undergo a repeat ultrasound in five to seven days.

The investigation findings were as follows - beta hCG on the day of surgery: 8700 IU/ml; beta hCG one week after surgery: 176 IU/ml; beta hCG one month after surgery: <2.00 IU/ml; hemoglobin (Hb) on the day of surgery: 12.3 g/dl; Hb on the following day, after one unit of packed cells was transfused: 9.4 g/dl.

Ultrasound at admission showed a heterogeneous mass in the right adnexa and a large amount of free fluid in the abdomen and pelvis, suggestive of a massive hemoperitoneum. The conclusion was a right ruptured ectopic gestation with hemoperitoneum.

Differential diagnosis

In a patient with a history of a previous ectopic who presents with all signs of an acute abdomen with severe pain, signs of significant blood loss, combined with a positive urine pregnancy test and an ultrasound examination suggestive of an adnexal mass with gross hemoperitoneum, the first diagnosis to be assumed must be an ectopic pregnancy. However, one should remember that a similar picture can also be associated with early intrauterine pregnancy and a bleeding or ruptured corpus luteum. It may also be seen in an early miscarriage and bleeding from another source if there is a history of trauma. Sometimes, this history of trauma may be difficult to elicit, especially if it is a case of domestic violence and the perpetrating partner is still a part of the patient’s life.

One should also consider other causes of abdominal pain, such as acute appendicitis, severe urinary tract infection (UTI), renal colic, ureteric calculus, torsion of the ovary containing the corpus luteum, torsion of an ovarian cyst, and acute pelvic inflammatory disease, to name a few. Any of these may present with or without a pregnancy.

Treatment

The patient was hemodynamically unstable when she returned to the hospital and, as the ultrasound suggested a hemoperitoneum and a left ectopic pregnancy, an emergency laparoscopy was performed. Significantly, the patient had a history of salpingectomy five months prior for a ruptured tubal gestation, and her left fallopian tube had been removed laparoscopically at that time.

On laparoscopy, a massive hemoperitoneum with clots and blood in the pelvis and the abdominal cavity was identified. On performing initial suction, the stump of the fallopian tube that had been previously removed was found to be bleeding profusely and chorionic tissue was seen protruding from it. The other fallopian tube was found to be normal with a few insignificant, small paratubal cysts. In an attempt to stop the blood loss, bipolar cautery was used and, in the process, the sac was extruded and picked up later by suction. The bleeding stopped and, due to the unstable condition of the patient, cornual resection was not performed.

Beta hCG on the day of surgery was 8700 IU/ml. The patient received one unit of packed cells after the surgery. A follow-up beta hCG, one week later, was 176 IU/ml. She recovered well from the procedure.

Outcome and follow-up

Beta hCG was done after one month and had fallen to less than 2.00 IU/ml. An ultrasound was also performed and found to be completely normal. The patient was pain-free and was able to resume her normal activities.

## Discussion

The documented incidence of ectopic pregnancy is 1-2% of all pregnancies. However, this statistic may be an underrepresentation as the cases of patients who receive treatment in an office setting may often go unreported [3]. Ectopic pregnancies, especially early and unruptured ones, often present a diagnostic dilemma. When a patient is seen with a positive urinary hCG and no intrauterine pregnancy is identified, all efforts should be made to exclude an ectopic pregnancy.

Our patient was first seen with amenorrhoea, positive urine hCG, and an inconclusive ultrasound that simply showed a thickened endometrium. At this point, such a case would generally be classified as a pregnancy of an unknown location [3]. If she had not been temporarily lost to follow-up at this stage, she would have benefited from a serum hCG level test to see whether it was in the discriminatory zone [4]. Even if a definite diagnosis could not be established, at the very least it could have helped to identify a patient that needed close follow-up with a repeat serum hCG and transvaginal ultrasound examination. In such a scenario, medical management with methotrexate may have helped her avoid the morbidity and trauma associated with the blood loss and emergency surgery [5]. This patient, unfortunately, did not receive the benefit of an early diagnosis, and presented about 10 days after the initial positive urine hCG test, with evidence of a hemoperitoneum.

The patient had a past history of a ruptured right tubal ectopic pregnancy six months prior to the current episode, which had been treated with laparoscopy and right salpingectomy. This history immediately increased her risk of having another ectopic [6]. She presented with severe lower abdominal pain and tenderness. She also had signs of hemodynamic instability such as dyspnoea and orthopnoea. On ultrasound, there was evidence of hemoperitoneum, and an adnexal mass was seen on the right side. No intrauterine pregnancy was identified and a presumptive diagnosis of a right ruptured ectopic pregnancy was made.

Due to the instability, pain, and hemoperitoneum, an emergency laparoscopy was performed. On laparoscopy, the remnant of the left tube that had been removed on the previous occasion was found to be bleeding profusely and chorionic tissue was seen protruding from it. This supports previous findings in the literature that ipsilateral salpingectomy predisposes to an interstitial pregnancy [7].

Even though the maternal mortality rate from ectopic pregnancies and their complications is decreasing, that from interstitial pregnancies remains much higher [7]. This has been attributed to (a) a high risk of rupture, and (b) more severe bleeding at a comparatively early period of gestation, due to less distensibility of the tube in this region and the anastomosis of the uterine and ovarian vessels leading to increased vascularity [8].

The mechanism of occurrence of these pregnancies in tubal remnants following an ipsilateral salpingectomy seems to be either recanalization of the remnant with peritoneal transmigration of the ovum, or internal transmigration [9]. This leads us to ponder as to whether anything can be done at the time of the initial salpingectomy to prevent a future pregnancy in the remnant. Strong evidence is lacking to support cornual resection in every ruptured ectopic as a means of prevention of interstitial pregnancy. A simple salpingectomy may be preferable to cornual resection as cornual resection is associated with interstitial pregnancies almost as often as a simple salpingectomy and is a much more morbid procedure [9]. Some authors have suggested minimizing the remaining length, along with peritonizing the cornual incision [10]. On the other hand, it may be impossible to prevent these, as small islands of tubal epithelium are seen in the cornual regions of the endometrium; even removing large areas of the cornua as prophylaxis may not prevent the occurrence of an interstitial pregnancy in a tubal remnant, especially when transuterine internal migration of the ovum is a possibility [9].

## Conclusions

An ectopic pregnancy in a tubal remnant is a rare condition. When it does occur, it leads to severe hemorrhage due to its location if it ruptures. It is equally amenable to medical management if diagnosed early, as has been shown in several previous reports. It is much more difficult to diagnose interstitial pregnancies than other ectopic pregnancies, as was seen in our case. In spite of the established criteria for ultrasound diagnosis such as an eccentric sac, in our case, the interstitial pregnancy was missed. The features that helped our team to decide on the laparoscopy were the features that were strongly suggestive of a ruptured ectopic, such as the hemoperitoneum on ultrasound, signs of hemodynamic instability, and the positive urine hCG test.

Therefore, although ultrasound is an invaluable tool in reaching a diagnosis, it is always a good practice to consider the patient's clinical features and history in their entirety.
